# High yield expression in a recombinant *E. coli *of a codon optimized chicken anemia virus capsid protein VP1 useful for vaccine development

**DOI:** 10.1186/1475-2859-10-56

**Published:** 2011-07-23

**Authors:** Meng-Shiou Lee, You-Cheng Hseu, Guan-Hua Lai, Wen-Te Chang, Hsi-Jien Chen, Chi-Hung Huang, Meng-Shiunn Lee, Min-Ying Wang, Jung-Yie Kao, Bang-Jau You, Wen- Hsin Lin, Yi-Yang Lien, Ming-Kuem Lin

**Affiliations:** 1 School of Chinese Pharmaceutical Sciences and Chinese Medicine Resources, China Medical University, Taichung, Taiwan; 2 Dept. of Cosmeceutics, College of Pharmacy, China Medical University, Taichung, Taiwan; 3 Graduate Institute of Biotechnology, College of Agriculture and Natural Resources, National Chung Hsing University, Taichung, Taiwan; 4 Dept. of Safety, Health and Environmental Engineering, Mingchi University of Technology, Taipei, Taiwan; 5 Graduate School of Biotechnology, Hung kuang University, Taichung, Taiwan; 6 Department of Medical Research, Tung's Taichung MetroHarbor Hospital, Taichung,Taiwan; 7 Institute of Biochemistry, College of Life Science, National Chung Hsing University, Taichung, Taiwan; 8 School of Pharmacy Undergraduate Program, Master Degree Program, Ph.D Program, China Medical University, Taichung, Taiwan; 9 Dept. of Veterinary Medicine, National Pingtung University of Science and Technology, Pingtung, Taiwan

## Abstract

**Background:**

Chicken anemia virus (CAV), the causative agent chicken anemia, is the only member of the genus *Gyrovirus *of the *Circoviridae *family. CAV is an immune suppressive virus and causes anemia, lymph organ atrophy and immunodeficiency. The production and biochemical characterization of VP1 protein and its use in a subunit vaccine or as part of a diagnostic kit would be useful to CAV infection prevention.

**Results:**

Significantly increased expression of the recombinant full-length VP1 capsid protein from chicken anemia virus was demonstrated using an *E. coli *expression system. The VP1 gene was cloned into various different expression vectors and then these were expressed in a number of different *E. coli *strains. The expression of CAV VP1 in *E. coli *was significantly increased when VP1 was fused with GST protein rather than a His-tag. By optimizing the various rare amino acid codons within the N-terminus of the VP1 protein, the expression level of the VP1 protein in *E. coli *BL21(DE3)-pLysS was further increased significantly. The highest protein expression level obtained was 17.5 g/L per liter of bacterial culture after induction with 0.1 mM IPTG for 2 h. After purification by GST affinity chromatography, the purified full-length VP1 protein produced in this way was demonstrated to have good antigenicity and was able to be recognized by CAV-positive chicken serum in an ELISA assay.

**Conclusions:**

Purified recombinant VP1 protein with the gene's codons optimized in the N-terminal region has potential as chimeric protein that, when expressed in *E. coli*, may be useful in the future for the development of subunit vaccines and diagnostic tests.

## Background

Chicken anemia virus (CAV), is the causative agent of chicken anemia disease. The disease results in severe anemia, lymph organ atrophy and immunosuppression [1-3]. CAV is the sole member of the genus *Gyrovirus *of the *Circoviridae *family. Histopathological studies have also shown that CAV infection leads to aplasia of the bone marrow and the destruction of T lymphoid tissue in young chickens [4,5]. When chicks are infected with CAV, the mortality rate is often as high as 55% and morbidity rates of 80% have been reported [6]. Therefore, worldwide, CAV is an economically important veterinary virus that can seriously affect the poultry industry.

VP1 protein (51 kDa) is the sole structural protein found within the CAV capsid. At a very late stage of the virus life cycle, the assembled virus particles created by VP1 protein spread into various other tissues and organs in the chicken, such as the thymus, spleen and liver [4]. Immunogenicity studies have shown that VP1 allows the elicitation of host-produced virus neutralizing antibodies [7]. Thus, VP1 is thought to be a good candidate for use as an immunogen when developing subunit vaccines and diagnostic kits [7,8]. Up to the present, a number of different expression systems have been used to produce VP1 protein, including *E. coli*, baculovirus-insect cells and plant cells [9-12]. However, production of the recombinant full-length VP1 protein has generally been unsuccessful because of a failure to express a span of amino acids at the N-terminus of the VP1 protein that is highly rich in arginine residues [9,11]. Furthermore, VP1 has been proposed to be cytotoxic in an *E. coli *expression system [9]. Once the N-terminus of VP1 is deleted, protein expression is improved significantly [11]. Nevertheless, the N-terminus of VP1 may still be involved in eliciting neutralizing antibodies because it contains some functional epitopes. Thus, there is a need to overcome the difficulties that have been encountered during production of full-length VP1 protein using an *E. coli *expression system. If successful, it would not only allow the efficient development of a subunit vaccine that is active against chicken anemia virus infection but the recombinant protein will also be potentially useful when developing diagnostic kits for the clinical detection of CAV infection.

As can be seen from the above information, *E. coli *has a number of disadvantages and limitations when producing CAV VP1 protein. However, the expression of VP1 protein in *E. coli *is still an attractive alternative to the current production system when assessed with respect to cost, time and operational considerations. In this study, we investigated the effect on expression of VP1 of changes in codon usage for various amino acids within the N-terminus of VP1 gene; this is because these amino acids are encoded by codons that are rarely used by *E. coli*. The codons within the N-terminus of the VP1 protein were optimized using prediction software and changed to the preferred codon usage for *E. coli*. In addition, VP1 was cloned into the expression vector, pGEX-4T-1, in order to create a glutathione-S-transferase (GST) fusion protein with the N-terminus of VP1. Expression of the modified VP1 genes was examined in various *E. coli *strains and changes in CAV VP1 protein expression determined. To the best of our knowledge, the yield of *E. coli *expressed recombinant VP1 in this study, after codon optimization of the N-terminal region, is the highest known to date.

## Results

### GST fusion tag enhances the expression of recombinant CAV VP1 protein in *E. coli*

**Figure 1:**
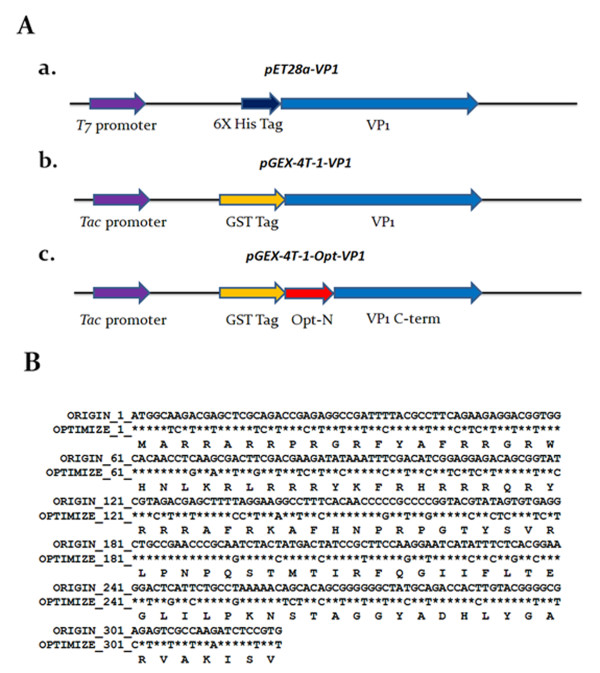
**Schematic diagram of the constructs used for CVA VP1 protein expression**. (A) Schematic representation of the VP1 protein variants and expression vectors used in this study. The designations of the VP1 protein and its expression vectors are indicated, a, b and c. The first two constructs, a and b, contained the full-length VP1 gene cloned into vectors pET28a and pGEX-4T-1, for expression of VP1 protein with a six-histidine (6 × His) tag and glutathione-s-transferase (GST) tag at its N-terminus, respectively. Construct c contained VP1 gene with codon-optimized at its N-terminus; this was derived from construct b by replacing the rare codons in the area 1-321 bp without altering amino acid sequence. The N-terminal codon-optimized VP1 gene, opt-N, was fused with C-terminal domain of VP1 gene (VP1 C-term) by overlapping PCR, and then cloned into pGEX-4T-1. (B) Sequence comparison between the WT VP1 and Opt VP1 gene. The nucleotide sequences were compared between the original VP1 gene (WT VP1) and the sequence of codon-optimized VP1 gene (Opt VP1) over the N-terminal region. An asterisk (*) represents the fact that the aligned nucleotides are identical.

**Figure 2:**
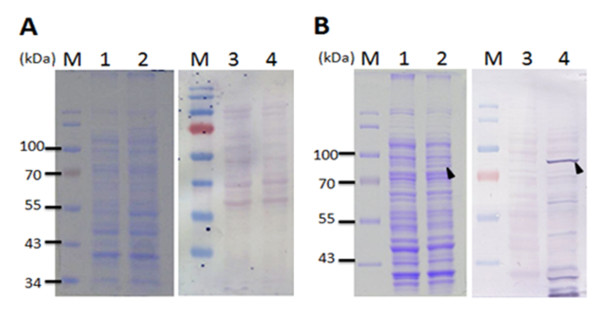
**VP1 protein expression in *E. coli *using various expression vectors**. The VP1 protein expression in *E. coli *BL21(DE3) using the expression vectors, pET28a (A) and pGEX-4T-1 (B) was analyzed by SDS-PAGE and Western-blotting. Anti-His tag and anti-GST tag monoclonal antibody were respectively used for the recognition of differently tagged VP1 proteins expressed by the different expression vectors used. Lane M, pre-stained protein marker; lane 1 and lane 3, before IPTG induction; lane 2 and 4, after IPTG induction for 4 h cultivation. The solid triangle pinpoints the expressed VP1 protein.

To investigate the effect of fusion tags on the expression of full-length VP1 protein of CAV in *E. coli *expression systems, the cDNA of VP1 was cloned into the pET-28a and pGEX-4T-1 vectors to obtain His tagged and GST tagged proteins. These constructs were designated to be pET28a-VP1 and pGEX-4T-1-VP1, respectively, and shown in Figure 1A. Using these two constructs, the expression of full-length VP1 protein in *E. coli *BL21(DE3) was examined. As shown in Figure 2, the full-length VP1 protein obtained from whole cell lysate was only expressed to any obvious extent in the recombinant *E. coli *harboring pGEX-4T-1-VP1 after IPTG induction for 4 h (Figure 2B, SDS-PAGE and Western-blot). The approximately 78 kDa GST-VP1 protein was recognized by monoclonal anti-GST antibody (Figure 2B, lane 4 of Western-blot). The induced control cell lysate containing pGEX-4T-1-VP1 plasmid without IPTG induction was not showed the presence of an extra band at 78 kDa (Figure 2B, lane 1 of SDS-PAGE, lane 3 of Western-blot). In contrast, VP1 was almost undetectable by Western-blot assay when anti-His tag antibodies (Figure 2A, lane 2 of SDS-PAGE and lane 4 of Western-blot) were used on the cell lysate from IPTG induced recombinant *E. coli *harboring pET28a-VP1. These results illustrated that the GST fusion tag improved protein expression significantly and allowed detectable amounts of intact full-length CAV VP1 protein to be produced in *E. coli*.

### Growth profile and expression levels of recombinant GST-VP1 protein in various different *E. coli *strains

**Figure 3:**
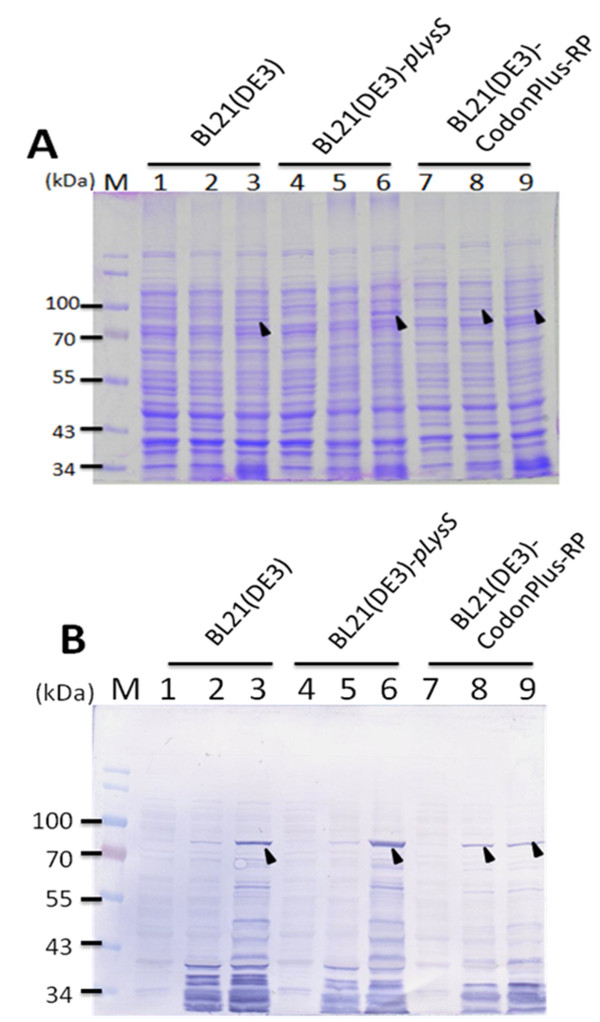
**Expression of recombinant GST-VP1 protein in three different *E. coli *strains**. The GST-VP1 protein expression in three *E. coli *strains, BL21(DE3), BL21-CodonPlus(DE3)-RP and BL21(DE3)-pLysS all containing pGEX-4T-1-VP1 was examined. The GST-VP1 protein was examined and detected using SDS-PAGE (A) and Western-blotting (B). Anti-GST tag monoclonal antibody was used to recognize the VP1 protein. Lane M, pre-stained protein marker; lane 1, 4 and 7, before IPTG induction; lane 2, 5 and 8, after IPTG induction for 1 hrs cultivation; lane 3, 6 and 9, after IPTG induction for 4 h cultivation. The solid triangle pinpoints the expressed VP1 protein.

**Figure 4:**
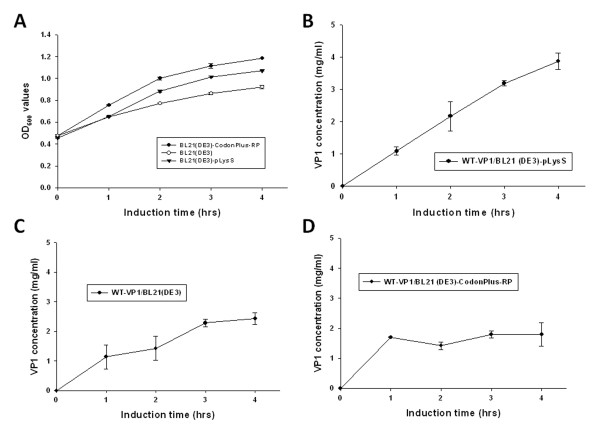
**Growth kinetics and production profiles of GST-VP1 protein in three *E. coli *strains**. (A) Growth profile of BL21(DE3), BL21-CodonPlus(DE3)-RP and BL21(DE3)-pLysS in LB medium post-induction. The production profiles of the three *E. coli *strains, (B)BL21(DE3)-pLysS, (C)BL21(DE3) and (D)BL21-CodonPlus(DE3)-RP containing pGEX-4T-1-VP1 are shown over time course after IPTG induction.

To further address the expression level of GST-VP1 protein in *E. coli*, three *E. coli *strains, BL21(DE3), BL21-CodonPlus(DE3)-RP and BL21(DE3)-pLysS were transformed with pGEX-4T-1-VP1 and used for protein induction and expression. Figures 3A and 3B demonstrate the protein expression pattern for identical amounts of whole cell lysate across the three *E. coli *strains, analyzed by SDS-PAGE and Western blotting. All three *E. coli *strains were able to express bands at the expected molecular mass of GST-VP1 protein after IPTG induction for 4 h (Figure 3A). Densitometric analysis of the blots showed that total expressed GST-VP1 protein, both soluble and insoluble, from BL21(DE3)-pLysS was one to two folds greater than the amounts produced by BL21-CodonPlus(DE3)-RP and BL21(DE3) (Figure 3B). The quantitative yields of GST-VP1 protein from BL21(DE3), BL21(DE3)-pLysS and BL21-CodonPlus(DE3)-RP, were induced for 1-4 h, and presented in Figure 4B, C and 4D, respectively. The amount of GST-VP1 protein produced by the three different strains was almost identical at 1 h of IPTG induction at 1.14 mg/ml for BL21(DE3), 1.08 mg/ml for BL21(DE3)-pLysS and 1.71 mg/ml for BL21-CodonPlus(DE3)-RP. After 2 h of induction, the expression level of GST-VP1 in BL21(DE3) and BL21(DE3)-pLysS both rose steadily, but had slowed down in BL21-CodonPlus(DE3)-RP (Figure 4B, C and 4D). The expression of GST-VP1 in BL21(DE3)-pLysS continued to increase significantly in BL21(DE3) over 2-4 h induction range but remained steady for BL21-CodonPlus(DE3)-RP (Figure 4B, C and 4D). These strains also show significant differences in their growth profiles. The optical density with 600 nm wave length (OD_600_) value reached by BL21-CodonPlus(DE3)-RP is higher than those of BL21(DE3)-pLysS and BL21(DE3) after IPTG induction even though GST-VP1 production is less (Figure 4A). Therefore, it can be concluded that BL21(DE3)-pLysS is the optimal host strain for producing full-length VP1 protein.

### Optimization of codon usage enhances the expression of recombinant CAV VP1 protein in *E. coli*

**Figure 5:**
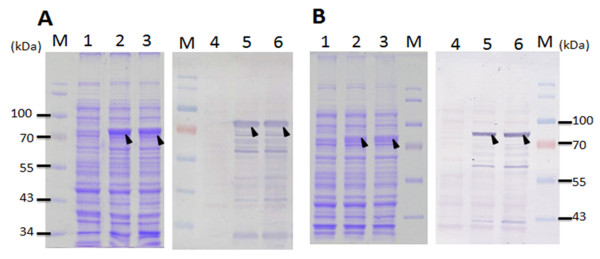
**GST-opt-VP1 protein expression in *E. coli *BL21(DE3)-pLysS (A) and BL21(DE3) (B)**. GST-opt-VP1 protein expression in *E. coli *BL21(DE3) and BL21(DE3)-pLysS was demonstrated and analyzed by SDS-PAGE and Western-blotting. Anti-GST tag monoclonal antibody was used to recognize VP1 protein when different expression vectors were used. Lane M, pre-stained protein marker; lane 1 and 4, before IPTG induction; lane 2 and 5, after IPTG induction for 1 h cultivation; lane 3 and 6, after IPTG induction for 4 h cultivation. The solid triangle pinpoints the expressed VP1 protein.

**Figure 6:**
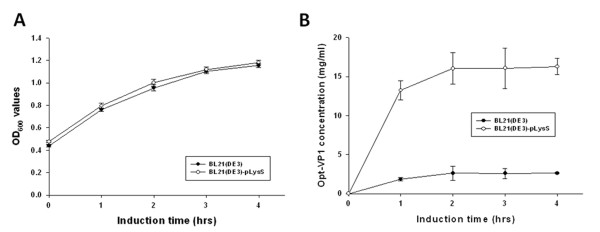
**Growth kinetics and production profiles of GST-opt-VP1 protein in *E. coli *BL21(DE3)-pLysS and BL21(DE3)**. (A) Growth profile of BL21(DE3) and BL21(DE3)-pLysS in LB medium during post-induction. (B) The production profiles of the *E. coli *strains, (B)BL21(DE3)-pLysS, and BL21(DE3) containing pGEX-4T-1-Opt-VP1 are shown after induction.

Based on the results shown in Figure 2B, we concluded that the expression of the full-length VP1 protein in *E. coli *was improved by fusing a GST tag to the N-terminus of VP1 protein. However, the expression of full-length VP1 protein in *E. coli *still gave a relatively low yield of GST-VP1 even when the optimal host strain BL21(DE3)-pLysS was used (Figure 4B). To further enhance the expression level of VP1, the codon usage of the VP1 gene was explored and optimized. To this end the CAV wild type VP1 gene was analyzed by PSORT II prediction http://psort.ims.u-tokyo.ac.jp/. The first 107 amino acid residues at the N-terminus of VP1 were found to contain many basic amino acid residues such as arginine and lysine (Figure 1B). The codons for these amino acids within the N-terminus of VP1 were then explored using the GenScript rare codon analysis tool http://www.genscript.com/index.html (Figure 1B). Clusters of rare codons are present in the 5' end of VP1 gene and therefore the CAV VP1 gene was optimized from base pairs 1-321 to *E. coli*'s preferred codons as selected by GeneOptimizer software. This was done without altering the amino acid sequence to be over-expressed in *E. coil*, which is confirmed by Figure 1B. The 321 bp DNA fragment from the N-terminus of VP1 protein was engineered to have the rare codons replaced by *E. coli*'s favored codons (AGA/CGA/CGG to CGT (R), CCC to CCG (P), CTC/CTT/CTT to CTG (L), GGA/GGG to GGT (G), ACT/ACA to ACC (T), CAA to CAG (Q) and ATA to ATC (I)) (Figure 1B). The new codon usage produced a better balance with respect to *E. coli *as indicated by the GeneOptimizer software. The new N-terminal codon optimized VP1 gene regions was assembled with the C-terminus of the VP1 gene to give an intact open reading frame using an overlapping PCR strategy. The new codon-optimized VP1 gene denoted opt-VP1 was then cloned into pGEX-4T-1 to give pGEX-4T-1-optVP1. This was then used for protein expression. As shown in Figure 5, the protein expression pattern of whole cell lysate in the *E. coli *strains BL21(DE3)-pLysS and BL21(DE3) were analyzed by SDS-PAGE and Western blotting. It was found that GST-opt-VP1 protein was successfully expressed after IPTG induction. The GST-opt-VP1 protein migrated at a expect molecular weight of 78 kDa in both BL21(DE3)-pLysS and BL21(DE3) and protein identity was confirmed by Western blotting using a monoclonal anti-GST antibody (Figure 5A and 5B, right panel). The quantitative yields for the GST-opt-VP1 protein in BL21(DE3)-pLysS reached to 17.5 mg/ml on the same optical density (OD) of cultures represents a 4.6 fold increase over wild type GST-VP1 expression (3.87 mg/ml) (Figure 6B and 4B). The expression of GST-opt-VP1 protein in BL21(DE3)-pLysS was 6 fold higher than the GST-opt-VP1 protein expression in BL21(DE3) (2.60 mg/ml), both after 4 h IPTG induction. No significant difference in the growth profiles of the two strains was observed post-induction (Figure 6A). Thus, in addition to improving the yield of full-length CAV VP1 protein using GST fusion, we have also further improved expression by codon preference optimization without altering the amino acid sequence.

### Purification of recombinant GST-opt-VP1 protein using GST affinity chromatography

**Figure 7:**
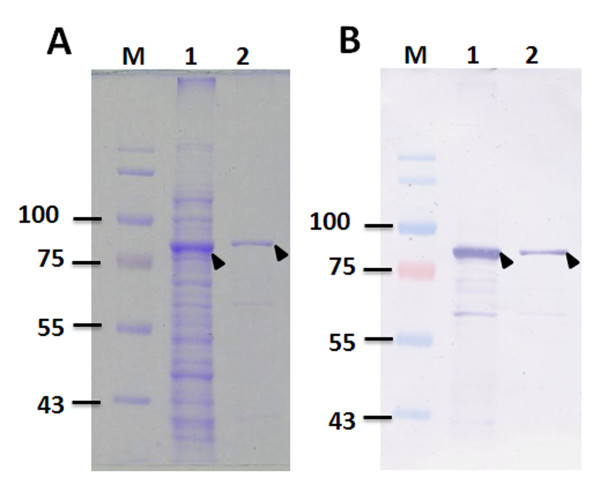
**Distribution of *E. coli*-expressed GST-opt-VP1 protein in the soluble and insoluble factions**. SDS-PAGE (A) and Western-blotting (B) were performed to examine the solubility of the *E. coli*-expressed VP1 protein. Anti-GST tag monoclonal antibody was used to recognize the VP1 protein. Lane M, pre-stained protein marker; lane 1, soluble fraction; lane 2, insoluble fraction.

**Figure 8:**
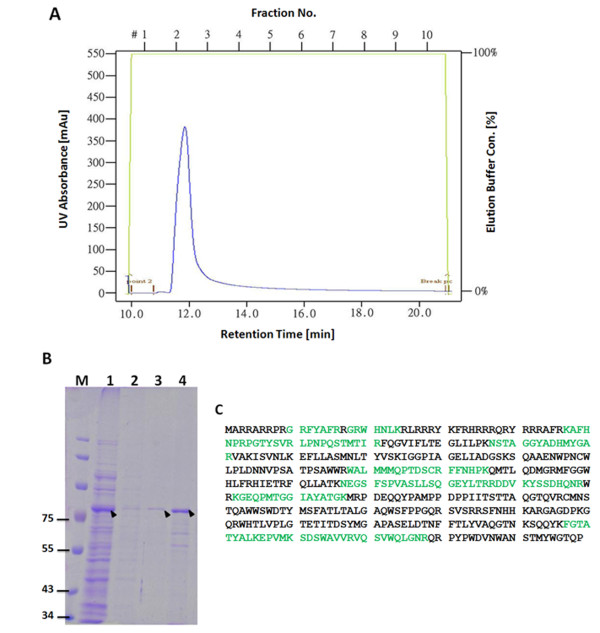
**Purification of recombinant GST-opt-VP1 protein**. (A) Chromatographic profile of GST-opt-VP1 protein eluted from a GSTrap FF affinity column. The cytosolic extract of *E. coli *strain BL21(DE3)-pLysS expressing GST-opt-VP1 protein was loaded into the GSTrap FF column and the bound protein was eluted with elution buffer as described in Material and Methods. (B) SDS-PAGE analysis of the eluted fractions collected from the GSTrap FF affinity column. Lane M, pre-stained protein marker; lane 1, flow through; lane 2, fraction obtained after column washing, lane 3 and 4, eluted fraction 1 and 2, respectively, collecting after column elution. (C) Identity of the GST-opt-VP1 protein determined by MALDI-TOF. The bold letters represent actual amino acid matches.

To purify GST-opt-VP1, the solubility of the expressed GST-opt-VP1 was examined. The supernatant and pellet obtained from a sonicated cell suspension was used. SDS-PAGE showed that approximately 80% of the GST-VP1 was soluble and this confirmed by Western blot using monoclonal anti-GST antibody (Figure 7A, B). Purification of the *E. coli*-expressed GST-opt-VP1 protein then proceeded using a GST affinity column. After affinity chromatography purification, the eluted soluble GST-opt-VP1 protein was monitored spectrophotometrically at OD_280 _and then separated by SDS-PAGE. A typical elution profile of the protein fraction collected from a GST column is shown in Figure 8A. Fraction 2 contains the most eluted protein from the GST column, has a significant absorbent peak at OD_280 _and was eluted at 12 min. The specific 78 kDa band eluted in fraction 2 was almost purified to homogenicity (Figure 8B, lane 4). The purified GST-opt-VP1 protein was then examined by MALDI-TOF (Figure 8C). Eight peptides from GST-opt-VP1 protein were identified after trypsin digestion and these demonstrated good alignment and a high score with the predicted protein. The longest peptide fragment of VP1, FGTATYALKEPVMLSDAWAVVRVQSVWQLGNR, consists of 32 amino acid residues (Figure 8C) and overall the fragments covered 34% of the published amino acid sequence of VP1 (Accession No. P54088) was obtained without any miss-match. These MALDI-TOF results confirmed that the purified 78 kDa protein was GST-opt-VP1 and that the *E. coli *preferred codon usage optimization within the VP1 gene was not seem to have altered the amino acid sequence of VP1 (Figure 8C; Figure 8B, lane 4)

### Application of recombinant CAV VP1 protein to an indirect ELISA

**Figure 9:**
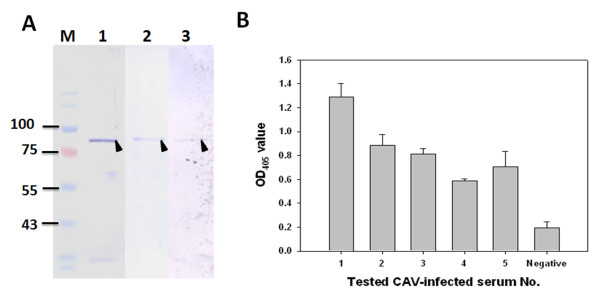
**Antigenicity of the expressed recombinant GST-opt-VP1 protein using CAV-infected chicken serum and Western blot analysis (A)**. Lane M, pre-stained protein marker; lane 1, 2 and 3, purified GST-opt-VP1 protein was respectively recognized by anti-GST mAb, anti-VP1 mAb E3 and CAV-infected positive chicken serum. Reactivity of the chicken serum with the recombinant GST-opt-VP1 protein was also evaluated by indirect ELISA (B). Five groups of CAV-infected positive chicken sera and one group of negative sera samples were used in a GST-opt-VP1 based ELISA. Five sera samples are involved in each group. These sera had been all identified as negative or positive using a commercial ELISA kit purchased from the IDEXX laboratory.

The recombinant full-length VP1 is a potent antigen that could be used to develop a diagnostic kit. In this context, the antigenicity of the recombinant GST-opt-VP1 protein was explored. The GST column-purified GST-opt-VP1 protein was found to exhibit good antigenicity when CAV-positive serum was used to recognize the antigen (Figure 9A, lane 3). The protein identity of GST-opt-VP1 was also examined by Western blotting using a monoclonal anti-GST antibody and monoclonal anti-VP1 E3 antibodies (Figure 9A, lane 1 and 2). The results indicated that *E. coli*-expressed GST-opt-VP1 protein retains considerable antigenicity and can be recognized by anti-CAV antibodies. In addition, to evaluate the possibility of developing a diagnostic kit, the *E. coli*-expressed GST-opt-VP1 protein was used as a coating antigen to set up a GST-opt-VP1 based indirect ELISA. As illustrated in Figure 9B, two hundred folds diluted CAV-negative and CAV-positive specific chicken sera were obtained from an experimental farm and used to represent different antigenicity responses against the GST-opt-VP1 protein using an ELISA approach; the results were measured at OD_405_. Purified GST-opt-VP1 protein showed higher antigenicity with the CAV-positive specific chicken sera (Sera No. 1-5) than with the CAV-negative chicken sera and there was a significant difference between the OD_405 _values for the CAV-positive and CAV-negative sera (*p *< 0.01). Next a cut-off value of 0.3 (Mean ± 2 fold standard deviation) was determined using the CAV-negative sera and this was used to define a positive threshold. Based on this criterion, the OD_405 _values of all CAV-positive sera were higher than this cut-off value. This indicates that CAV positive/negative sera can be successfully discriminated using the recombinant protein and this threshold. Thus, the purified GST-opt-VP1 protein shows considerable antigenic potential and able to pinpoint sera from chickens that are infected with CAV and separate these from the sera of uninfected chickens.

## Discussion

In this study, the specific aim was to establish a prokaryotic expression system for the production of full-length recombinant CAV VP1 protein. A prokaryotic expression system has several advantages when expressing a heterologous recombinant protein; these include cost-effectiveness, time-saving, ease of production and the fact that the antigenic characterization of recombinant protein are easier to confirm. Therefore, *E. coli *expression systems are the most used approaches when screening for the expression of a foreign protein [15]. Indeed, among above points are many of the critical factors associated with choosing a suitable production system for the development of a subunit vaccine and diagnostic kit. However, in previous studies, there have many difficulties when expressing full-length CAV VP1 protein using *E. coli *as the expression host [9,11]. In addition to VP1 protein expression, proteolysis in *E. coli *also seems to occur resulting in the degradation of the VP1 protein [9]. Although an N-terminus deleted VP1 has been successfully expressed in *E. coli*, nevertheless, the use of a truncated form of the CAV VP1 protein might hampers its usefulness in serological tests or as a subunit vaccine. Thus, the problems caused by the lack of a truly effective system for producing VP1 protein still needed to be overcome.

Using Genscript OptimumGene™ bioinformatic software, the rare codons in *E. coli *that exist in the wild-type CAV VP1 gene were analyzed. These amino acid residues include arginine, leucine, proline and lysine, which are common in the N-terminus region of the VP1 protein (1-321 bp). Rosenberg *et al *proposed that the abundance of rare codon near the 5'-end of the gene might affect the efficiency of protein translation and this suggested the present approach to increasing expression of the full-length CAV protein [16].

Both of porcine circovirus (PCV) and CAV are members of the circovirus family. PCV also has similar basic amino acid residues rich in rare codons at the N-terminus of the capsid protein and this also results in decreased protein expression [17]. However, Liu *et al *successfully overcame this problem by fusing a maltose-binding protein (MBP)-His × 8 tag to the PCV capsid protein for expressing and the resulting fusion protein in *E. coli *[18]. Why the presence of a MBP-His × 8 tag improves protein expression remains unclear, but one possibility is improved protein solubility [19]. Similarly, in the present study, the addition of a GST tag to the VP1 protein improved expression in *E. coli *significantly compared to a His × 6 tag (Figure 2B). Thus it would seem that some fusion tags are able to promote better expression in *E. coli *than other tags, perhaps by aiding the correct folding of their fused partner protein [19]. This report is the first to show that full-length recombinant CAV VP1 protein can be expressed successfully in *E. coli *by adding a GST tag.

Presently, it had been recognized that VP1 is a sole structural protein. This protein is not only an immunogen for used as vaccine candidate but also suitable applied as a coating antigen of ELISA for diagnostic purpose [7,8,10]. However, there has no any prokaryotic expression system which successful employed for production of full-length VP1 of CAV. In addition, the full-length VP1 of CAV involved all of the epitopes which can elicit virus neutralizing antibodies of the host. For this study, the full-length VP1 protein was soluble with 80% of total expressed protein, and retained the antigenic characteristics of the native VP1 (Figure 7 and 9B). Thus, it implied that the most important immunodomain, the VP1 of the CAV capsid, can highly yield produced herein, and will be used conveniently for developing recombinant vaccine in the other further studies.

Although the full-length of recombinant CAV VP1 protein was able to be expressed in *E. coli *by fusing the VP1 protein with a GST tag, the yield of GST-VP1 remained relatively low at (approximately 3.78 mg/ml (Figure 4B). This may be a result of poor protein stability, the toxicity of CAV VP1 protein or others factor including perhaps rare codons within the VP1 gene [9,11]. When the full-length VP1 protein was expressed in BL21 (DE3), BL21 (DE3)-pLysS and BL21 (DE3)-CodonPlus-RP, the highest level of the full-length VP1 protein occurred with in BL21 (DE3)-pLysS. This perhaps indicates that stain BL21 (DE3)-pLysS might be more tolerant of any cytotoxicity. BL21 (DE3)-CodonPlus-RP was able to reach an expression level of 1.71 mg/ml at 1 h post-induction, which is about 30% higher than in BL21 (DE3) and BL21 (DE3)-pLysS under the same conditions (Figure 4B, C and 4D). One reason for the increase in full-length VP1 expression by BL21 (DE3)-CodonPlus-RP cells might be the presence of extra copies of the tRNA^*argU, proL *^genes. However, such over-expression might exhaust endogenous energy supplies, which is deleterious to the cells, especially when they are induced with IPTG induction. In this context, it was found that IPTG induction of over 2 h, when the bacterial cells were grown at 37°C, failed to increase the yield of VP1 further to any significant degree. Also noteworthy is the fact that the growth profile of BL21 (DE3)-CodonPlus-RP during the course of expression of GST-VP1 was not changed (Figure 4A). This phenomenon might cause by a balancing of the steady-state growth condition of the strains with yield, which suggests that the strain had reached its maximum possible growth rate for expressing full-length CAB VP1 [20].

In such circumstances, it seems likely that optimization of codon bias would be a useful strategy to improve the expression of full-length CAV VP1 protein. By engineering the foreign gene to use *E. coli*'s preferentially used codons; this will improve translation efficiency without the need to supply extra copies of the rare tRNA genes [21-23]. When the rare codons within the N-terminus of the VP1 gene were optimized, expression of full-length optVP1 protein in BL21 (DE3)-pLysS significantly increased (Figure 5B, 6B). The comparably higher yield of optVP1 to wild type VP1 or any other published expression system after the rare codons had been optimized within the N-terminus of VP1 protein and using BL21 (DE3)-pLysS, was approximately 4.6 fold greater than for wild type VP1 at 1 h post-induction. Notably, an increase in induction to greater than 2 hours was not further increased the accumulation of optVP1 protein. The limit may be the protein burden within the *E. coli *cells, which eventually leads to growth arrest (Figure 6A, B). However, using a fed-batch based cultivation system, this difficulty might be overcome and allow even higher yields of full-length VP1 protein to be obtained in the future.

## Conclusions

The expression of recombinant full-length VP1 protein of CAV using a prokaryotic system was established successfully and the yield was greatly improved by making various adjustments to the expression system. Optimization of codon usage within N-terminal region of VP1 protein was a most convenient and cost-effective way of increasing the expression of full-length CAV VP1 protein in *E. coli*. This paves the way for large-scale production of the full-length VP1 protein using this approach, which will allow the full-length VP1 to be used for the development of a subunit vaccine or a diagnostic test.

## Methods

### Bacterial strain and cells inoculation

BL21 (DE3) was obtained from Invitrogen Life Technologies (Carlsbad, CA). BL21-CodonPlus(DE3)-RP and BL21(DE3)-pLysS were purchased from Stratagene (La Jolla, CA). Overnight cultures (10 ml LB medium in 50 ml flasks) were grown at 37°C for strain activation. Then 0.5 ml of the overnight culture were inoculated into 50 ml LB medium and grown at 37°C for around 3 h by which time the optical density of culture had reach 0.5 of OD_600 _for cell transformation or protein expression.

### Plasmid construction

A 1350 bp of cDNA encoding the full-length CAV VP1 protein was cloned into pET28a (Novagen, Madison, WI) earlier (Figure 1A, panel a) (Lee et al., 2009) [11]. Using a specifically designed primer set, forward primer CAV VP1 F: 5'-GCGGAATTCATGGCAAGACGAGCTCGCAGA-3' and reverse primer CAV VP1-R: 5'-GGGCTCGAGTCAGGGCTGCGTCCCCCAGTA-3', respectively containing the *EcoR*I and *Xho*I sites, the full-length VP1 gene was amplified using the plasmid pET28a-VP1 as template DNA, and then cloned into the plasmid pGEX-4T-1 (GE healthcare, Piscataway, NJ), which was then designated to be pGEX-4T-1-VP1 (Figure 1A, panel b). This construct carries the VP1 gene flanked by the N-terminal glutathione-S-transferase (GST) tag and will express a GST-VP1 fusion protein. To generate the plasmid harboring the codon optimized nucleotide sequence, a codon optimized fragment encoding the first 107 amino acid residues of the N-terminus of the VP1 capsid protein was fused with the C-terminus of the VP1 protein using the 5' and 3' ends of above two DNA fragments and overlapping PCR [13]. The PCR used three different primer sets, Opt-F1 (5'-CCCGAATTCATGGCTCGTCGTGCTCGTCGT-3') and Opt-R1 (5'-CGCTAGCAGGAACTCTTTCAGGTT-3'), Opt-F2 (5'-AACCTGAAAGAGTTCCTGCTAGCG-3') and CAV VP1-R for the first amplification and then CAV VP1 F and VP1-R for the second amplification. The result was a fragment containing the entire cDNA, Opt-VP1, which was then ligated into pGEX-4T-1 and designated to be pGEX-4T-1-Opt-VP1 (Figure 1A, panel c). The constructed recombinant plasmids were transformed into One Shot^® ^Top10 (Invitrogen, CA) chemically competent *E. coli *for the maintenance of the recombinant plasmids and protein expression. Transformants that contained a gene of the correct size by PCR were then checked using restriction enzyme digestion and DNA sequence analysis.

### Codon optimization of cDNA of the N-terminus VP1 protein

The codons of the cDNA of the N-terminus of VP1 protein (321 bp) were optimized based on the codon preference of *E. coli *using Genscript OptimumGene™ designing software. The codon-optimized cDNA of the N-terminus of VP1 protein was synthesized by Genomics Biosci & Tech Co. (Taipei, Taiwan) and then assembled into the C-terminus of VP1 protein to give the entire VP1 gene using the approach described above [13].

### Expression of full-length VP1 protein after codon optimized in recombinant *E. coli*

The host *E. coli *strains BL-21(DE3), BL21-CodonPlus(DE3)-RP and BL21 (DE3)-pLysS have different recombinant constructions and these strains were used for protein induction and expression. The recombinant strains were grown in LB medium in the presence of kanamycin (50 μg/ml), ampicillin (50 μg/ml) or chloramphenicol (34 μg/ml) as appropriate at 37°C. When the culture had reached an optical density (OD_600_) of 0.5, isopropyl-β-D-thiogalactopyronoside (IPTG) at different concentrations was added to induce protein expression and then growth was continued for 4 h. After IPTG induction, samples of the cells were harvested using centrifugation for 10 min at 10,000 rpm. To determine the protein expression, the cell pellets were resuspended in 1xSDS-PAGE sample buffer and boiled for 5 min. The resulting lysates were then Western blotted after separation on a 12.5% SDS-PAGE. The expression level and concentration of the recombinant VP1 proteins were determined as described in a previous study [11].

### Purification of recombinant VP1 protein using GST affinity chromatography

To purify the recombinant VP1 proteins, the cells were spun down from 50 mL of culture supernatant and resuspended in GST resin binding buffer (140 mM NaCl, 2.7 mM KCl, 10 mM Na_2_HPO_4_, 1.8 mM KH_2_PO_4_, pH 7.3). They were sonicated on ice three times for 3 minutes with a 20% pulsed activity cycle (MISONIX Sonicator^® ^300). The lysate was then centrifuged for 10 min at 10,000 rpm to remove the cell debris. The resulting cell supernatant was injected into a GSTrap FF affinity column (GE healthcare, Piscataway, NJ) with 1 mL of GSTrap resin at a flow rate of 0.5 ml/min. The packed column was washed with a 10-fold volume of binding buffer, and then the bound proteins were eluted with elution buffer (50 mM Tris-HCl, 10 mM reduced glutathione, pH 8.0) at 1 ml/min. Each fraction consisted of 1 ml of elutant. The fractions were monitored at OD_280 _using the optical unit of a liquid chromatography system (AKTAprime plus, GE Healthcare BioScience AB, Uppsala, Sweden). A putative peak containing the recombinant CAV viral protein was identified and the eluant collected for analysis. The total protein concentration of each fraction was determined using a Micro BCA kit (Pierce, Rockford, IL) using bovine serum albumin as the reference protein. The purity of the protein sample was analyzed using aliquots of the fractions by 12.5% SDS-PAGE and Western-blotting with appropriate antibodies.

### Mass spectrometry

To confirm the identity of the recombinant GST-opt-VP1 protein directly expressed in *E. coli *or purified by GSTrap FF column, the proteins were separated by 12.5% SDS-PAGE. The relevant band was then cut out from the 12.5% SDS-PAGE gel after Coomassie blue staining and digested with trypsin. The resulting samples were subjected to the MALDI-TOF-MS mass spectrometry (ESI-QUAD-TOF) to allow amino acid sequence identification of the protein, as described in a previous study [11].

### ELISA assay

The antigenicity of *E. coli *expressed VP1 protein after purification was evaluated by ELISA assay. Briefly, 2 μg per well of diluted VP1 protein was coated onto a 96 wells plate for 1 h at 37°C. After washing, CAV-infected positive serum was added and reacted for one another hour at 37°C. Subsequently, following washing, secondary antibodies were added and this was followed by color development as described in a previous study [14].

## Competing interests

MSL, YYL and GHL are inventors on a patent submission entitled: a method for high yield production of chicken anemia virus VP1 capsid protein. The other authors declare no competing interests.

## Authors' contributions

MSL participated in this study design, performed the experiments and in the writing of the manuscript. GHL performed the experiments and participated in the construction of the plasmids. YYL participated in the experiments on protein antigenicity and GHL, CHH, MYW, BJY and JYK participated in the protein purification step. MKL and MSL^5 ^participated in the data analysis and the writing of the manuscript. HJC, YCH, WTC and WHL coordinated the study and participated in the writing of the manuscript. All authors read and approved the final manuscript.
